# Treatise on Diseases of the Skin

**Published:** 1833-10

**Authors:** 


*Treatise on Diseases of the Skin. Founded on new Researches in Pathological Anatomy and Physiology.* By P. Rayer, D.M.P. *Translated from the French,* by W. B. Dickinson, Member of the Royal College of Surgeons. London, 1833. 8vo. pp. 400.

Dr. Rayer's treatise is evidently the work of a physician of industry and judgment, and his classification of cutaneous diseases, which we subjoin, shows considerable ingenuity:

"SECTION I. DISEASES OF THE SKIN.

| | |
|---|---|
| CHAPTER I. *Inflammations of the Skin.* | 1°. *Exanthematous:* Rubeola, roseola, scarlatina, urticaria, erythema, erysipelas.<br>2°. *Bullous:* Vesication, ampullæ, pemphigus, rupia, zona.<br>3°. *Vesiculous:* Herpes, psora, eczema, miliaria.<br>4°. *Pustulous:* Varicella, variola, vaccinia, vaccinella, ecthyma, cuperosa, mentagra, impetigo, tinea, artificial pustules.<br>5°. *Furunculous:* Hordeolum, furuncle, anthrax.<br>6°. *Papulous:* Strophulus, lichen, prurigo.<br>7°. *Tuberculous:* Lupus, cancer, elephantiasis of the Greeks.<br>8°. *Squamous:* Lepra, psoriasis, pityriasis.<br>9°. *Linear:* Fissures.<br>10°. *Gangrenous:* Malign pustule, carbuncle of the plague.<br>11°. *Multiform:* Burns, frost-bite, syphilitic eruptions. |
| CHAPTER II. *Cutaneous and Subcutaneous Congestions and Hæmorrhages.* | Cyanosis, vibices, petechia, purpura hemorrhagica, ecchymosis, dermatorrhagia. |
| CHAPTER III. *Neuroses of the Skin.* | Exaltation, diminution, abolition of the sensibility of the skin, without appreciable alteration in the texture of this membrane. |
| CHAPTER IV. *Alterations in the Colour of the Skin.* | *Decoloration*: Leucopathia (Partial; General); Chlorosis<br>*Accidental Colorations*: Ephelis, lentigo, chloasma, meladermis, icterus, *nœvus maculosus,* bronze tint produced by the internal use of lunar caustic. |

| | |
|---|---|
| CHAPTER V.<br>*Morbid Secretions.* | Ephidrosis, acne, folliculous tumours. |
| CHAPTER VI.<br>*Defects of Conformation and Texture; Hypertrophies, and Accidental Productions.* | Distention of the skin; cicatrices, vegetations, nævus hematodes, subcutaneous vascular tumours; warts, pearly granulations; corns, ichthyosis, horny appendages. |

"SECTION II.

ALLTERATIONS OF THE APPENDAGES OF THE SKIN.

| | |
|---|---|
| CHAPTER I.<br>*Alterations of the Nails, and of the Skin which produces them.* | Onyxis; increased growth of the nails; spots, change of colour, fall, desquamation, reproduction, &c. of the nails. |
| CHAPTER II.<br>*Alterations of the Hair, and of the Follicles which produce it.* | Inflammation of the bulbs of the hair; accidental colorations, canities; alopecia; matting of the hair; plica; accidental pilous tissue. |

"SECTION III.

FOREIGN BODIES OBSERVED ON THE SURFACE, OR IN THE SUBSTANCE OF THE SKIN.

| | |
|---|---|
| *Inanimate.* | Dirt, dirt of the scalp of new-born children; inorganic matters, artificial colorations. |
| *Animate.* | *Pediculus humani corporis; P. capitis, P. pubis; pulex irritans, P. penétrans; acarus scabiei; æstrus; gordius.* |

"SECTION IV.

DISEASES PRIMARILY FOREIGN TO THE SKIN, BUT WHICH SOMETIMES PRODUCE PECULIAR ALTERATIONS IN THIS MEMBRANE.

Elephantiasis of the Arabs."

We quote the following passage, because it is interesting in itself, and treats of a subject which has been omitted by Bateman.

"*Bronzed Tint of the Skin, produced by the Nitrate of Silver.*

"§ 655. Nitrate of silver, employed internally now for some years, in the treatment of certain nervous diseases, and particularly epilepsy, sometimes produces a bronzed tint of the skin, analogous to that of mulattos, and which may increase to blackness.

"§ 656. This coloration appears to have been first observed by Swediaur. 'A protestant minister,' says he, 'labouring under obstruction of the liver, took, by the advice of an empiric, a solution of arg. nit. Having continued its use for several months, the skin changed insensibly, and at last became nearly black. This colour remained for several years, and then began to diminish. J. A. Albers, of Brême, prescribed, in 1801, the arg. nit. for an epileptic woman, about thirty years of age. This patient, relieved by the remedy, continued its use for three years and a half. Towards the end of the last year, she being pregnant, the skin became blueish, particularly of the face, neck, hands, and nails; the sclerotic was also coloured. The blue tint increased at the approach of the menstrual period: the colour of the blood was natural, and she was in other respects quite healthy; and, despite of various measures had recourse to, the skin retained its blue colour. Struck with the singularity of this phenomenon, Albers inquired if other practitioners had observed it. Reimar, of Hamburg, wrote to him that he had met with two cases. Professor Rudolphi said that a similar result had been observed by a physician at Greifswalde. Doctors Schleiden and Chaufepié have communicated three cases of this coloration. Dr. Roget, of London, prescribed lunar caustic for a young woman affected with epilepsy, and she continued its use for four or five months; and he remarked, after the suspension of the remedy, that the tongue and pharynx assumed a blackish-brown shade. At the end of some months, a darkness was observed beneath the eyes, and successively on different parts of the body. This change was permanent, and in no way influenced by the menstrual discharge. Three similar cases are mentioned by Butini, in his work on the internal use of the arg. nit. Professor Sementini has published a memoir on the same subject. M. Planche, in giving an analysis of this work, says that he saw, in 1817, in Guy's Hospital, a woman, seventy years of age, the whole of whose body was of a deep blue tint, after being treated by the nitrate of silver. Lastly, I have myself seen this change in the colour of the skin, in four epileptics who had been submitted to the influence of this remedy.

"§ 657. I saw at the Bicêtre two other epileptics, treated unsuccessfully with the nitrate of silver, and who presented this dark tint of the skin. One of them had this morbid alteration very marked, particularly on the hands and face; it was fainter on the parts kept constantly covered. This patient had several cicatrices of the same tint as the skin. The mucous membrane of the tongue and the conjunctivæ were of a similar colour as the tegument; the hair and nails had undergone no change.

"§ 658. When diffused throughout the animal structure, does the nitrate of silver undergo any particular modification, or does it operate some peculiar alteration on the mucous body? Or, again, does it meet on the surface of the body with nitrate of potass, and thus become transformed into an insoluble muriate, as some authors have imagined?

"§ 659. The alteration of colour in the skin caused by this salt cannot be confounded with any other change in the pigment; it is very different even from the black colour produced by rubbing the lapis infernalis over the skin.

"§ 660. This bronzed tint of the skin has not, as yet, yielded to any of the means employed for its removal. It generally diminishes after some years' duration; but I am not aware that its entire disappearance has ever been effected. Perhaps this coloration might be dissipated by the employment of some stimulating baths; since Badeley has ascertained that, after the application of blisters, the skin assumes its natural tint." (P. 303.)

In the above quotation, for Brême, we ought to have had Bremen.

Mr. Dickinson's translation is tolerable, though far too literal.

